# The Growth and Trends in Cancer‐Induced Cachexia Research: A Bibliometric and AI‐Guided Visualization Analysis

**DOI:** 10.1002/cnr2.70690

**Published:** 2026-10-01

**Authors:** Thomas M. O'Connell, Radhika Gedela, Fabrizio Pin

**Affiliations:** ^1^ Department of Otolaryngology, Head & Neck Surgery Indiana University School of Medicine Indianapolis Indiana USA; ^2^ Indiana Center for Musculoskeletal Health Indiana University School of Medicine Indianapolis Indiana USA; ^3^ Luddy School of Informatics, Computing and Engineering Indiana University Indianapolis Indianapolis Indiana USA; ^4^ Department of Anatomy, Cell Biology and Physiology Indiana University School of Medicine Indianapolis Indiana USA

**Keywords:** bibliometric analysis, burst analysis, cachexia, cancer, cancer‐induced cachexia, research trends, trend analysis

## Abstract

**Background:**

Over the past several decades, advances in basic and clinical research have increasingly established cancer‐induced cachexia as a distinct pathophysiological syndrome that warrants targeted therapeutic approaches alongside conventional anticancer treatments. Despite its high prevalence and substantial impact on morbidity, mortality, and quality of life, the condition remains underrecognized and inadequately managed in clinical practice. Correspondingly, scientific activity in the field has expanded rapidly, spanning molecular mechanisms, clinical characterization, and emerging interventional strategies.

**Methods:**

This bibliometric analysis aims to provide a comprehensive, quantitative overview of evolving research in cancer‐induced cachexia over the past 25 years. We conducted a bibliometric analysis of cancer cachexia research published between 2001 and 2025 using the Web of Science Core Collection. A total of 5775 English‐language primary research articles were retrieved using the search terms “cancer AND cachexia,” and including papers in English of the types articles, letters, and proceedings papers. Data were analyzed using the *Bibliometrix* R package and its web interface, BiblioShiny, to assess publication trends, author productivity, collaboration networks, and keyword co‐occurrence. Additional visualizations were generated using the R programming language and AI‐guided analyses to explore trends and bursts in the evolution of cachexia research.

**Results:**

Cancer cachexia publications increased at an annual rate of 6.7%, exceeding the overall growth rate of scientific literature. In terms of number of papers and citation indices, the Journal of Cachexia, Sarcopenia and Muscle is the dominant journal in the field. Research is characterized by high levels of international collaboration, though research networks remain geographically clustered. Keywords were analyzed using co‐occurrence networks to reveal specific connections and clusters of research topics. Trend analyses of keywords showed the progression of research in the areas of metabolism and the role of inflammatory mediators. Burst analysis revealed research “hotspots” over the last 25 years including studies of specific interventions such as immunotherapy, gastrointestinal cancers, and metabolic dysregulation.

**Conclusions:**

Cancer cachexia research has expanded significantly over the past two decades but remains limited in scale and fragmented across geographic and disciplinary boundaries. A shift toward investigating biological mechanisms and therapeutics indicates a maturing field with growing translational potential. Growing trends and recent research bursts in immune function and inflammation suggest an important role of these areas in further therapeutic development. Continued integration of clinical, nutritional, and molecular research will be essential to advance effective interventions. This bibliometric analysis offers a data‐driven foundation to inform future research priorities and collaboration strategies in cancer‐induced cachexia.

## 1 Introduction

Cancer cachexia is a complex metabolic syndrome characterized by severe body weight loss, muscle wasting, and systemic inflammation that impacts about a third of all cancer patients [1]. It is now recognized as a critical determinant of patient outcomes, influencing treatment tolerance, physical function, and quality of life. Despite its profound clinical implications, cancer cachexia has historically received limited attention relative to other cancer‐related complications. Only in recent decades has cachexia been increasingly acknowledged as an independent and urgent clinical problem, deserving of dedicated research and therapeutic development [2]. The interdisciplinary and multifactorial nature of cachexia, which spans molecular biology, clinical oncology, nutrition, and palliative care, has made it challenging to coalesce research efforts into a unified and translational agenda.

Bibliometric analysis offers a powerful approach to quantitatively assess the evolution, structure, and dynamics of scientific fields over time. By applying bibliometric techniques to the domain of cancer cachexia, we can identify key trends, influential contributors, collaborative patterns, and emerging research themes. These insights can help not only to map the intellectual landscape of the field but also to highlight gaps in knowledge and opportunities for future inquiry. There have been several published bibliometric analyses related to cachexia, but mostly focusing on the associations with aging and sarcopenia [3, 4, 5, 6, 7]. To our knowledge, this is the first comprehensive, longitudinal bibliometric analysis specifically focused on cancer cachexia.

In this study, we conducted a 25‐year bibliometric analysis of cancer cachexia literature from 2001 to 2025 using data derived from peer‐reviewed scientific publications indexed in Web of Science Core Collection (WOSCC). We examined publication trends, authorship and collaboration networks, journal impact, citation patterns, keyword co‐occurrence patterns, trend analysis, and burst analysis to assess the field's growth and intellectual structure. To help interpret these analyses, we enlisted AI models to cluster and annotate the keyword clusters. These analyses provide a detailed and data‐driven overview of how the field has evolved and where it may be headed. These findings are intended to inform researchers, clinicians, and policymakers interested in improving both the scientific understanding and clinical management of this devastating syndrome.

## 2 Methods

### 2.1 Data Collection and Filtration

A comprehensive bibliometric analysis of cancer cachexia was conducted using the Web of Science Core Collection (WOSCC) database (https://www.webofscience.com/wos/woscc/basic‐search). The literature retrieval was conducted on a single day, March 27th, 2026, to ensure dataset consistency and to capture a fixed snapshot of the database's contents. The search terms used were “cancer AND cachexia,” with the document types limited to articles, letters, or proceedings papers. Articles were also limited to those written in English. The publication period covered 2001–2025.

The search produced a total of 5775 articles, which were then exported in “plain text” format with the document type set to “full record and cited references.” This export included detailed information for each article, such as titles, authors, institutions, abstracts, keywords, keywords plus, publication dates, journals, and cited references. The plain text is available in Supporting Information S3 as is the Excel file S2 exported from the Bibliometrix platform (version 5.4.1) [8, 9].

An assessment of completeness for the data extraction was performed. Table S1 shows that there was complete retrieval of information on authors, document type, journal, language, publication year, science categories, title and total citation for all the records. Incomplete retrieval was found for affiliation (15, 0.26%), DOI (230, 3.98%), cited references (9, 0.16%), corresponding author (17, 0.29%), abstract (166, 2.87%), and keywords (1219, 21.11%) and keywords plus (206, 3.57%). Note that keywords plus are keywords generated by an algorithm that analyzes the titles and the titles of articles cited by the author [10].

### 2.2 Bibliometric Analysis

The bibliometric analysis was conducted using the BiblioShiny platform of the *Bibliometrix R package* to quantify research trends, including the identification of leading authors, institutions, and countries, as well as the analysis of citation patterns and keyword co‐occurrence [8, 9]. This tool facilitated a statistical approach to understanding the evolution and focus of cancer cachexia research over time.

Author keywords and Keywords Plus terms were standardized by converting terms to lowercase, trimming whitespace, and removing missing values. Co‐occurrence network analysis was carried out in the R programming language, version 4.6.1. For each publication, all unique pairwise keyword combinations were generated using combinatorial expansion, and repeated keyword pairs across publications were summed to produce weighted edges representing co‐occurrence frequency. The igraph package was used to generate the network including properties such as node degree and weight. Community structure was identified using the Louvain modularity optimization algorithm, which detects groups of keywords that co‐occur more frequently with each other than with the rest of the network. Networks were visualized using the ggplot and visNetwork packages with node size representing keyword frequency or degree and edge thickness representing co‐occurrence strength.

Naming of the co‐occurrence network communities was carried out using an AI‐assisted semantic labeling approach. For each precomputed community, the associated keyword terms were aggregated and provided to the GPT‐3.5‐turbo large language model (LLM) with instructions to generate a concise 2–5 word community name and a one‐sentence description summarizing the dominant biomedical theme. The model was prompted to base its interpretation on the provided keyword list, producing human‐readable labels for communities identified in the keyword network. To ensure reproducibility and consistency, a standardized prompt template was used across all communities, with fixed instructions, output formatting requirements, and domain‐specific context. Model parameters were held constant (e.g., temperature = 0) to minimize stochastic variation, and all outputs were generated in a single‐pass, automated workflow without manual intervention. This approach provides consistent, interpretable labels grounded in the semantic composition of each keyword community while maintaining a transparent and reproducible annotation process. Details of the R code, including system and user prompts are given in the Supporting Information S1 entitled, AI Prompt Code for Community and Cluster Naming.

Trend analysis was carried out in BiblioShiny which computes the frequency of the keyword across all documents along with the median year indicating the central time of the keyword's usage over time. The first and third quartiles are also computed to show the temporal distribution. Visualization of the data as dot plots was carried out in R.

Burst detection was performed using a keyword‐specific temporal enrichment approach implemented in R. For each keyword, annual frequencies were computed and compared to baseline usage across the full time series. Keywords with low overall frequency were excluded to reduce noise. For each remaining keyword, a baseline mean and standard deviation of yearly counts were calculated and burst years were defined as those in which the observed frequency exceeded a minimum count threshold and showed both a ≥ 2‐fold increase relative to the keyword's baseline mean and a standardized deviation (z‐score) ≥ 2. This approach prioritizes sustained and statistically elevated usage over isolated rare events, enabling identification of meaningful temporal surges in keyword activity.

For the trend and burst analyses, keyword clustering and annotation were performed using a combined embedding‐based and AI‐assisted approach. First, filtered keyword terms were converted into dense vector representations using SapBERT [11], a transformer‐based model trained on biomedical terminology, enabling semantically similar terms to be positioned proximally in high‐dimensional space. The resulting embeddings were clustered using the k‐means clustering algorithm (*k* = 3, nstart = 25) to group related keywords into distinct communities based on semantic similarity. As with the co‐occurrence network communities, the AI‐defined clusters were named using the same LLM‐guided approach used to annotate the co‐occurrence network communities described above.

## 3 Results

### 3.1 Publication Output and Growth

The bibliometric analysis on cancer cachexia covered a timespan from 2001 to 2025. A total of 5775 documents were collected from 1341 unique sources including articles, letters, and proceedings papers written in English. Figure 1 shows that the data fits an exponential growth curve with an *R* ^2^ value = 0.98. A linear fit of the log transformed publication data is shown in Figure S1 and shows an *R* ^2^ value for the linear model equal to 0.95. The results show an annual growth rate of 6.7%. The overall growth rate of scientific publications has recently been reported to be 4.1% with an annual doubling time of 17.3 years [12]. Using the “rule of 70” in statistics whereby the doubling time is approximated as 70/(annual growth rate %) [13] the doubling time for publications is about 10.4 years indicating that cancer cachexia is a rapidly expanding field.

**FIGURE 1:**
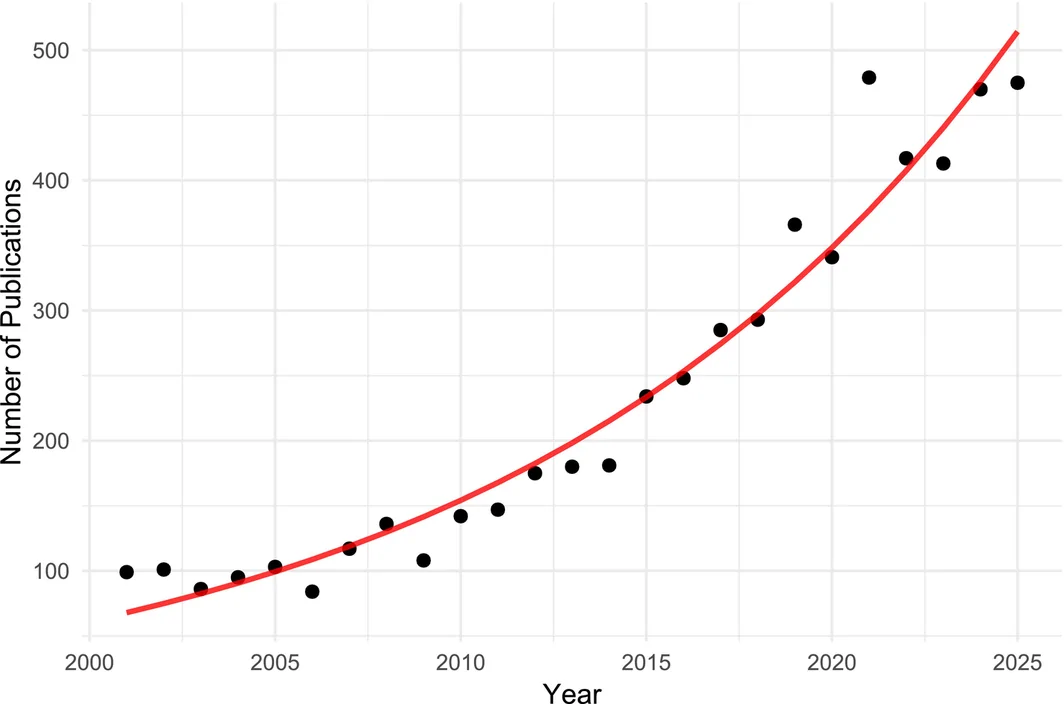
Annual scientific production between 2001 and 2025. Red line shows the fit to exponential function with *R* ^2^ value = 0.98.

The average age of documents in the dataset is 8.86 years, suggesting that a substantial portion of the research is relatively recent. On average, each document received 35.89 citations, reflecting the impact and relevance of studies in this field. Collectively, these documents cited 125 477 references, highlighting the extensive bibliographic foundation of cancer cachexia research.

### 3.2 Analysis of Journals

The production and impact of specific journals on the field is shown in Figure 2. The journals are evaluated based on the total number of papers, the number of locally cited sources, that is, the number of citations within all the publications in this dataset and lastly the local H‐index indicating the journal's overall productivity and impact among those in this dataset. The H‐index was calculated according to the method described by Hirsch, defined as the maximum number of publications (*h*) that have received at least *h* citations each [14]. In this figure, all the journals that were found in the top 10 for any of these three metrics are included in each of the plots, thus 18 journals are shown. The top 10 journals in terms of number of papers are shown in Figure 2A. The journals with the most locally cited sources are shown in Figure 2B and the journals with the highest impact as defined by the local H‐index are shown in Figure 2C. The Journal of Cachexia Sarcopenia and Muscle is clearly a dominant journal in this field being at the top of each of the metrics. It is interesting to note that the British Journal of Cancer is in the top 10 in both local citations and H‐index, but it not in the top 10 in numbers of papers. Also, the last two journals in the top ten in local H‐index are not in the top 10 in either number of papers or local citations.

**FIGURE 2:**
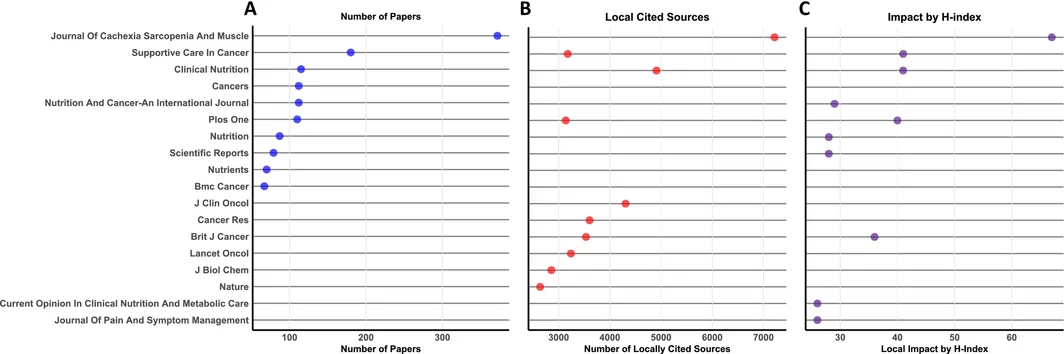
Analysis of journal output, citations and impact. Journals include any that are in the top 10 of number of papers (A), locally cited sources (B) or H‐index (C).

### 3.3 Analysis of Authors

A total of 31 545 authors contributed to this body of work. The collaboration level was high, with an average of 8.1 co‐authors per document and 21.4% of articles involving international co‐authorship, reflecting significant global collaboration.

The productivity and impact of specific investigators and research groups are evaluated by the number of papers, author local citations, and local H‐factor as shown in Figure 3. The superset of the top 25 authors in each of the three metrics is included and thus 43 authors are shown in the plots. The number of publications from the top 25 authors is shown in Figure 3A. The authors with the most locally cited sources are shown in Figure 3B and the authors with the highest local H‐index are shown in Figure 3C. Of the top 25 authors in terms of the number of papers, 11 are also in the top 25 in locally cited sources, and 19 are in the top 25 in local H‐index.

**FIGURE 3:**
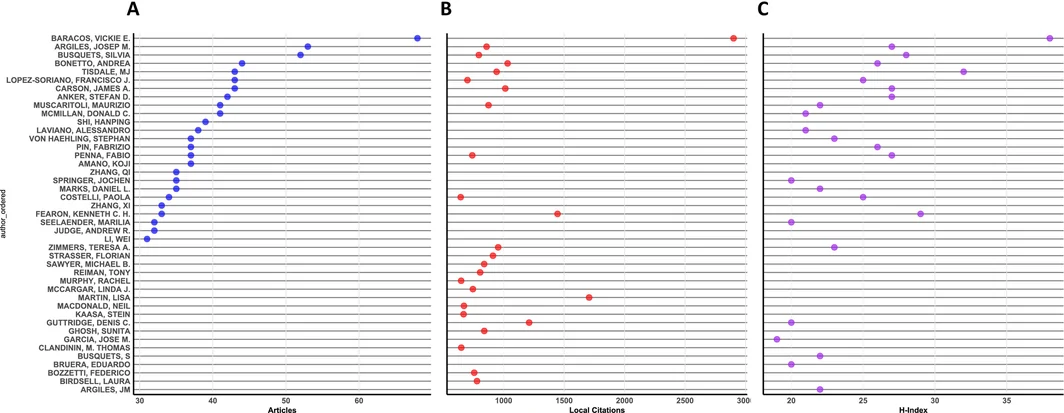
Analysis of author output, citations and impact. Authors include any that are in the top 10 in terms of numbers of papers (A), locally cited sources (B), and H‐index (C).

### 3.4 Analysis of Collaborations

The collaborative patterns of research are shown in Figure 4. The network of authors in Figure 4A has the author node sizes associated with the degree, that is, number of collaborative connections. The edges indicate co‐authorship with the weight of the edge indicating the number of times the authors have co‐authored a paper. For clarity only the top 50 authors in terms of network degree are included in the plot. Community analysis using the Louvain algorithm was applied to the network to identify communities of authors who collaborate frequently. A set of six communities were found with several showing distinct regional influences. The blue network is composed of predominantly Italian researchers, the purple network is mainly Asian researchers and the small green group is composed of four German researchers. The red network is more diverse and appears to be dominated by researchers who have collaborated with the prolific and highly cited author, Professor Baracos. It must be noted that these communities are not isolated, but rather highly interconnected showing that international collaborations are strong.

**FIGURE 4:**
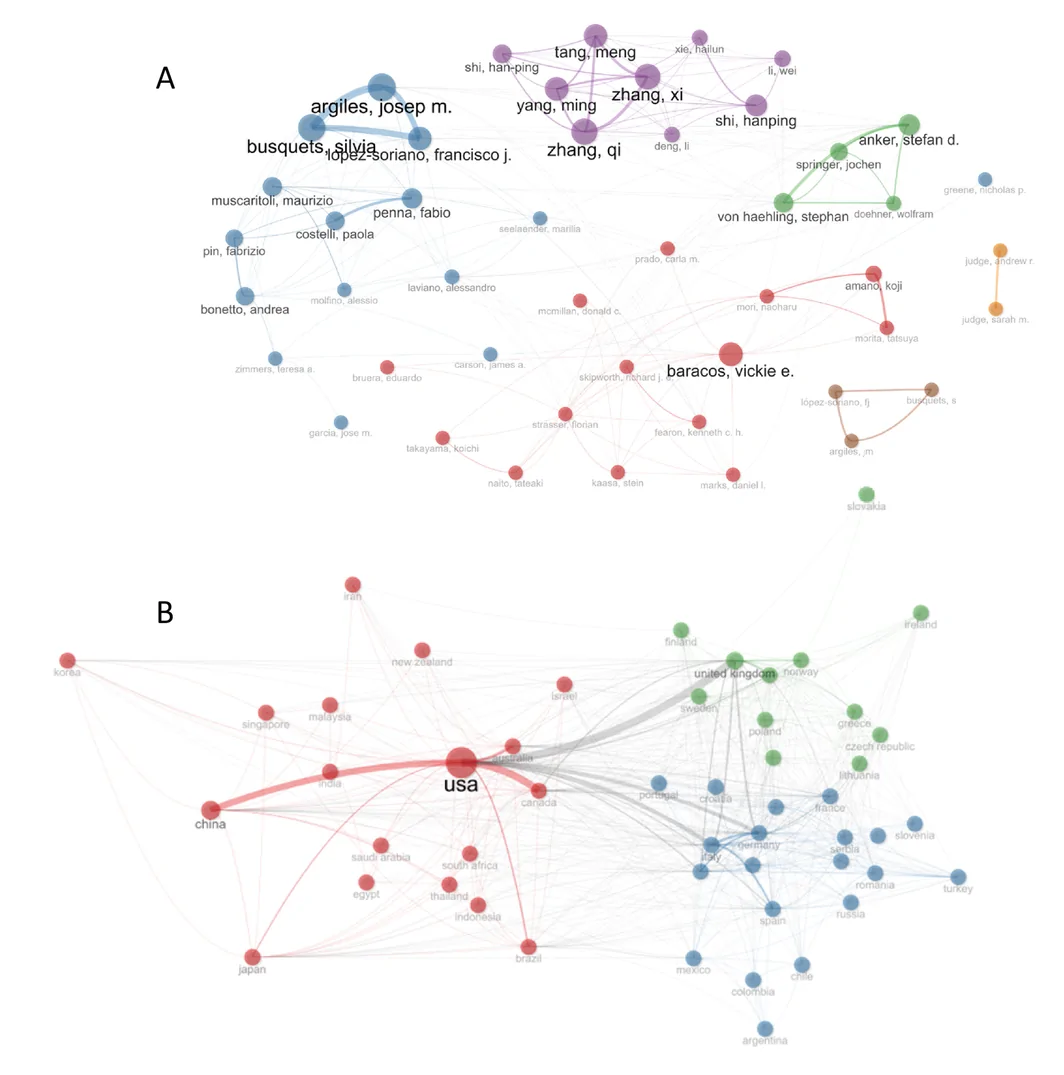
Collaboration network. (A) Co‐occurrence network is based on co‐authorship of papers and (B) co‐authorship of papers from different countries. Cluster analysis using the Louvain algorithm identifies distinct groups of authors and countries with extensive collaborations.

To further evaluate regional influences, Figure 4B was generated to show the number of papers with co‐authors from different countries. The Louvain algorithm was used to identify network communities. The blue and green networks are dominated by European countries while the red network is more diverse. This network contains the strong collaborations between the USA, China, and Canada. As with the co‐author network, the communities are highly interconnected, confirming the presence of strong global collaborations.

To evaluate the output of different countries several metrics are shown in Figure 5. The top 10 countries in terms of number of publications are shown in Figure 5A. The bars are split into two parts, including those with the corresponding author(s) from a single country and those with corresponding authors from multiple countries. Figure 5B shows the top 10 countries in terms of number of citations. Figure 5C shows the top 10 countries in terms of average citations per paper. Figure 5D compares the rank order of the countries by the categories. A few notable patterns emerge from this plot. The USA is at the top in terms of total paper and total citations and fourth in average citations per paper. China is 2nd and 3rd in total papers and citations but out of the top 10 in average citations per paper. Similarly, Japan is 3rd and 5th in total papers and citations but out of the top ten in average citations per paper. Countries that have lower total paper counts but higher total citations and average citations per paper include the UK and Canada.

**FIGURE 5:**
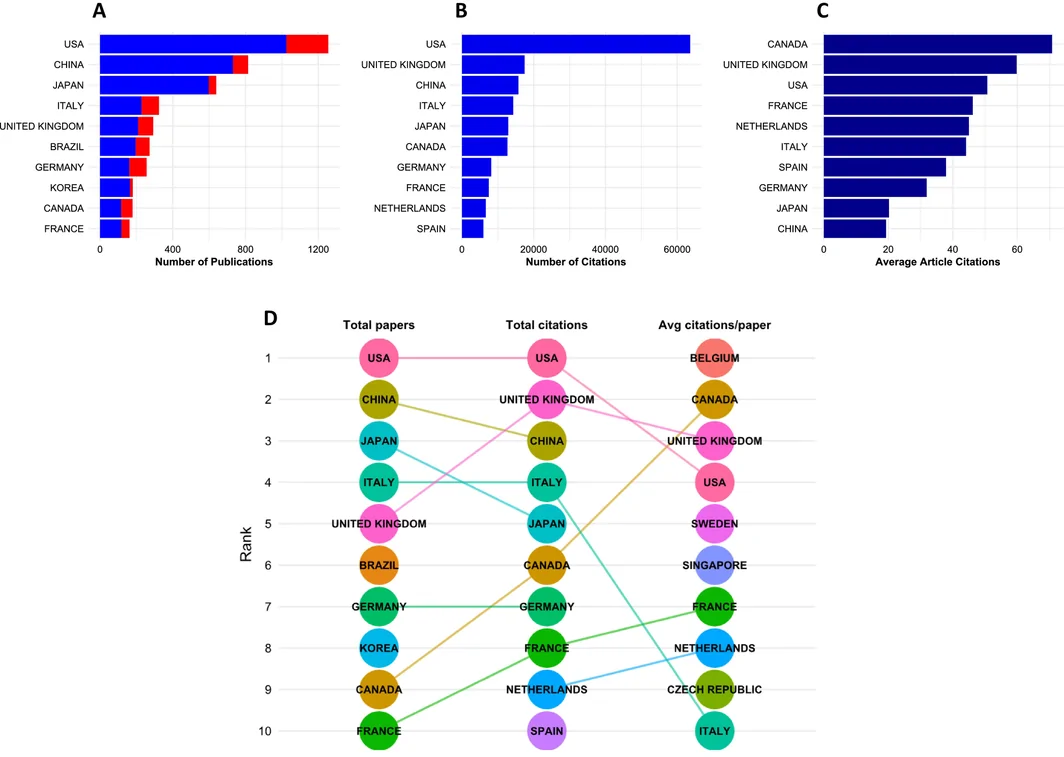
Country publication output in numbers of papers and citations. (A) The top 10 countries in terms of total number of papers with a corresponding author from that country. The red segment of the bars indicates those papers that also have authors from different countries. (B) The top 10 countries in terms of number of citations. (C) The top 10 countries in terms of average number of citations per article. (D) Slope chart showing how the ranking of individual countries changes across the three metrics.

### 3.5 Analysis of Citations

The impact of a paper on a field of study is often associated with the number of times the paper has been cited. A set of citation metrics for all 5775 articles are given in Table S2. This table includes the number of local citations, that is, those citations within this dataset and the global citations, that is, all citations including those outside this dataset. In addition, normalized versions of these metrics are included where the age of the paper is accounted for, thereby accounting for the time the paper has been available for citation. This is carried out by dividing the citation count by the number of years since publication, plus 1 to avoid dividing by zero. Figure 6 shows the papers that are in either the top 10 of normalized local or global citations. Of the top 10 locally cited papers, eight of them are associated with defining or managing cachexia and thus have broad interest in the field; four of these are also in the top 10 of the normalized global citations. Only 5 papers are in both the top of both local and global citations. At the bottom of the figure are several papers with rather high global citations indices, but very low local indices suggesting that the papers have high significance but have yet to be well integrated or recognized by the cachexia community. This pattern could suggest promising new areas of cachexia research.

**FIGURE 6:**
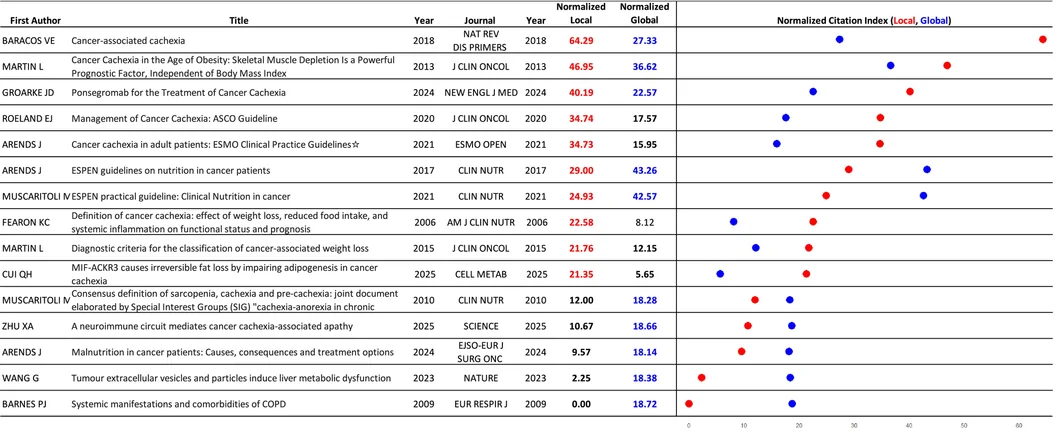
Top papers in terms of normalized local or global citations. The table section lists the papers that are in the top 10 by either the normalized local citations or normalized global citations. The dot plot maps these citation metrics.

### 3.6 Analysis of Keyword Co‐Occurrence and Trends

The full set of papers contains 66 922 keywords combining author‐provided keywords and Keywords Plus. Keywords Plus are algorithmically generated terms derived from the titles of cited references within WOSCC records and are intended to supplement author‐provided keywords [10]. As expected, many keywords appear in numerous articles leaving 13 594 unique words. The co‐occurrence network shows which keywords are frequently found in the same article suggesting a thematic connection. The full network would contain over 200 K edges, so the network analysis was limited to only those keywords that had a minimum of 10 connections. Figure 7 shows this co‐occurrence network composed of 88 nodes and 1597 edges. Very common terms including cachexia, cancer cachexia, cancer, skeletal muscle, weight loss, and expression were removed as they would be very high degree nodes that are too general to be very informative. Community analysis using the Louvain algorithm identified four distinct communities. To better understand and describe the nature of these communities, the keywords from each community were annotated using AI‐assisted semantic labeling. The results are shown in Table 1. More common terms like survival, sarcopenia, and chemotherapy are found in the center of the network with high degrees and corresponding large node sizes. At the edges of the network are more unique and specific terms. For example, the green nodes associated with the Muscle Metabolic Regulation community include a number of specific cytokines. The purple nodes in the Cancer Appetite Modulators community contain several different small molecule and peptide nodes including anamorelin, eicosapentaenoic acid, leptin, and ghrelin. The Cancer Care Management community contains keyword nodes such as nutrition, palliative care, fatigue, and quality of life.

**FIGURE 7:**
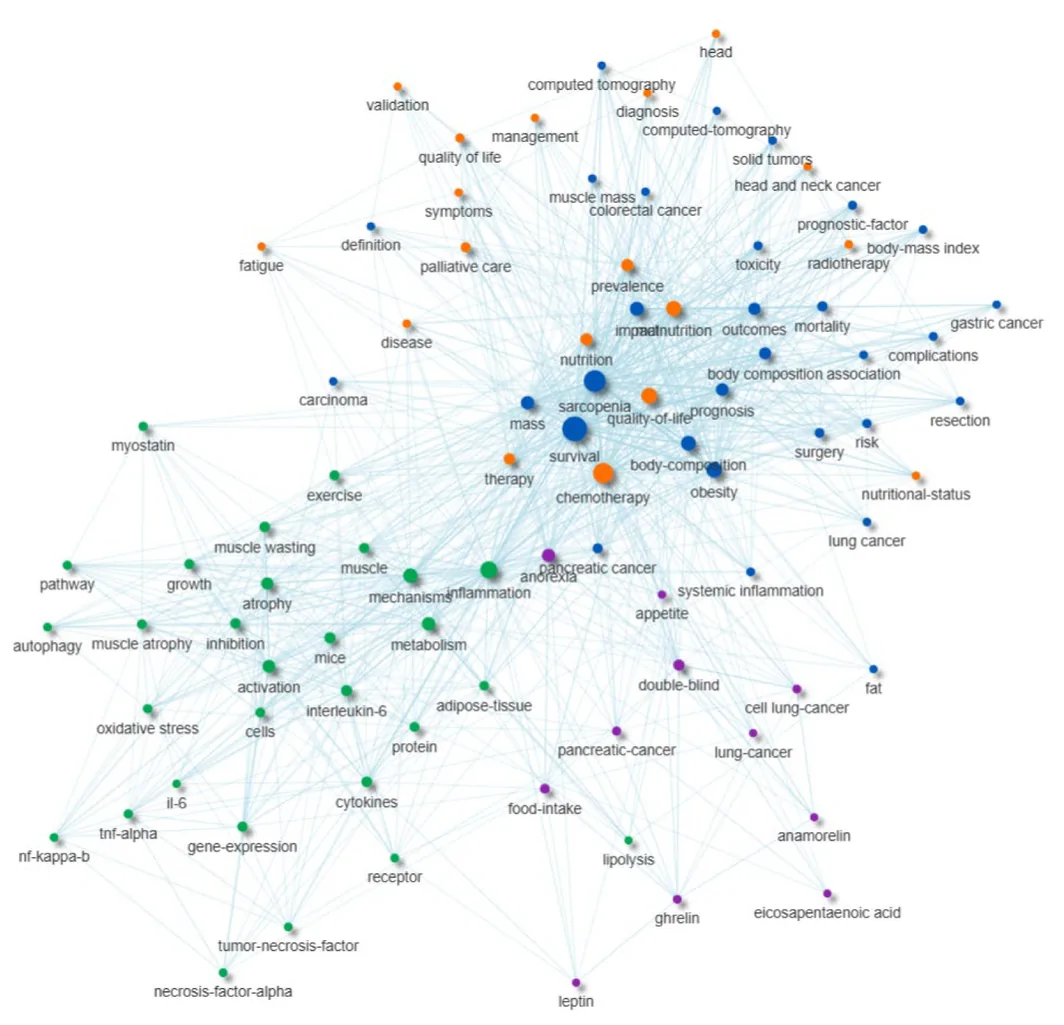
Keyword co‐occurrence network. The network includes keywords and Keywords Plus from 2001 to 2025. A threshold was set so that only keywords that had a minimum of 10 connections are shown. Network community analysis revealed four communities described in Table 1.

**TABLE 1:** AI guided naming and descriptions of the co‐occurrence network communities in Figure 7.

Community	Color	Name	Description
1	Blue	Cancer Prognosis & Body Composition	Focus on the impact of body composition on cancer prognosis, mortality, and treatment outcomes across various types of cancer, with a particular emphasis on sarcopenia and obesity.
2	Orange	Cancer Care Management	Related to cancer treatment including therapy, nutrition, quality of life, and symptom management.
*3*	*Green*	Muscle Metabolic Regulation	Related to the complex interplay between inflammation, metabolism, muscle wasting, and regulatory pathways involved in muscle homeostasis
4	Purple	Cancer Appetite Modulators	Related to various aspects of cancer and appetite regulation, such as pancreatic and lung cancers, as well as hormones like ghrelin and leptin, highlighting potential therapeutic targets for modulating appetite in cancer.

A trend analysis of the keywords is shown in Figure 8. A set of 73 keywords were included in the analysis that met a threshold of having a minimum word frequency of 5 and minimum words per year of 3. To simplify the visualization and add context to the analysis, keyword clustering and annotation were carried out using AI‐assisted embedding and LLM analysis. The number of clusters was set to three. When higher numbers of clusters were used, the results included large disparities in cluster size, for example, some clusters with dozens of keywords and others with only a few terms. Table 2 shows the names and descriptions of each of the three clusters. In the plots, the location of the dot indicates median of the frequency distribution of the keyword and the span of the line covers the time between the 1st to 3rd quartiles of the distribution. Dot size indicates the frequency of usage. The largest cluster in red focuses on metabolism and therapy impact. At the top of the plot are a number of very high frequency, and rather general terms such as cancer and skeletal muscle. Several terms such as muscle protein degradation, tissue protein‐turnover and ubiquitin‐dependent proteolysis all have median years and distributions before 2010 indicating that interest in these topics has waned. The inflammatory mediators cluster includes a number of cytokines, peptides, transcription factors and drugs. There is a progression of cytokines in the literature with a peak of research on interferon‐gamma in 2006. Peaks for the tumor‐necrosis factor and necrosis factor alpha are seen around 2008 and 2012. Interleukin‐6 has the highest frequency and spans a wide range centered around 2015. The most recent cytokine research appears to be on GDF‐15, a member of the TGF‐beta superfamily with a recent peak at 2025. Research on the orexigenic compounds, neuropeptide‐y and megestrol‐acetate peaked around 2005 and 2007 respectively. More recent research in appetite modulation is shown by the peaks in the keyword anamorelin around 2023 and the drug formulation of this compound, Ono‐7643 around 2024.

**FIGURE 8:**
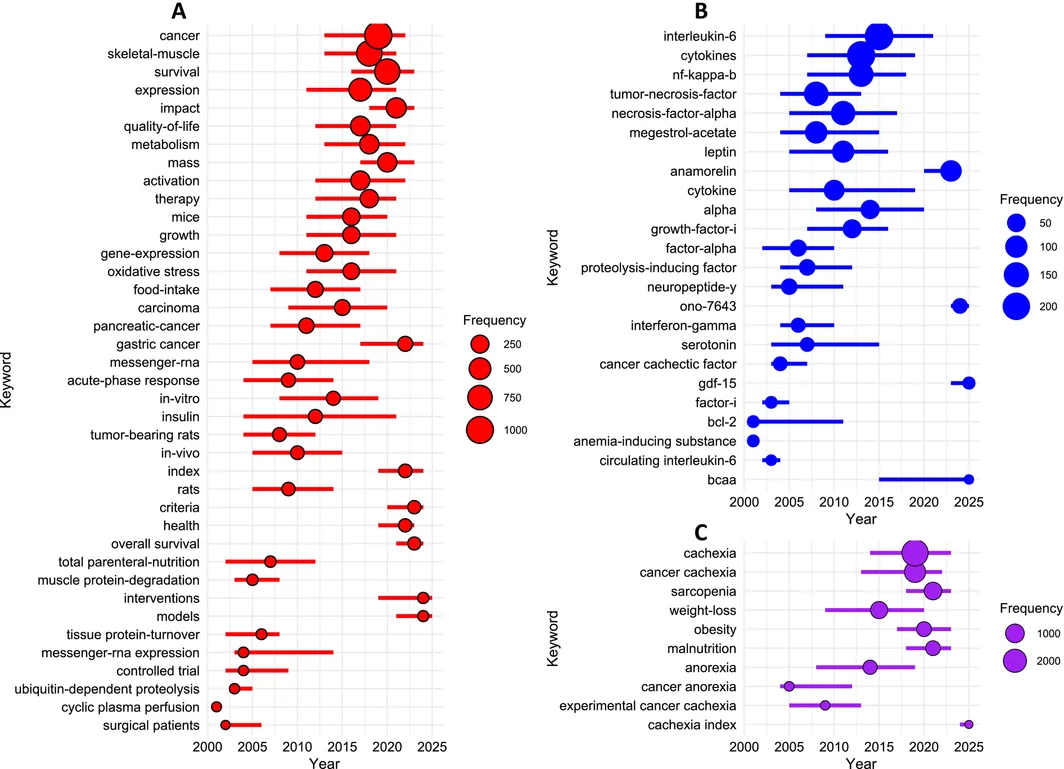
Trend Analysis of keywords. Keywords include those with a minimum word frequency of 5 and minimum words per year of 3. Cluster analysis was used to identify three groups of keywords using AI‐embedding and LLM analysis provided the names and descriptions of each cluster shown in Table 2. The dots are positioned at the median of the frequency distribution and the lines span the range from the 1st to 3rd quartile of the range.

**TABLE 2:** Names and descriptions of the AI‐guided clusters for the trend analysis in Figure 8.

Cluster	Panel	Name	Description
1	A	Cancer Metabolism and Therapy Impact	Research on the impact of metabolism and therapeutic interventions on cancer progression and survival outcomes, involving animal models and clinical trials.
2	B	Inflammatory Mediators	Related to various inflammatory mediators involved in cancer cachexia and anemia, including interleukin‐6, cytokines, and tumor necrosis factor
3	C	Cancer‐related Metabolic Disorders	Related to cancer‐induced metabolic disturbances such as cachexia, sarcopenia, anorexia, and weight loss

Another way to evaluate the research trajectories in cancer cachexia is to look for bursts in keyword usage suggesting a temporal research “hotspot.” The burst analysis computes annual frequencies of keywords and highlights those where the frequency showed an increase of greater than five‐fold over an estimated baseline usage. This threshold revealed 65 keywords that demonstrated at least one burst in usage. Figure 9 shows the result of this analysis as a temporal event tick plot where small lines indicate the occurrence of a keyword and clusters that comprise a burst are designated by boxes. As with the trend analysis, AI‐assisted embedding and LLM annotation were used to divide the 65 terms into three clusters. The names and descriptions of the clusters are shown in Table 3. It is interesting to note that the overlap in the keywords identified in the trend and burst analyses is quite small and includes only the following eight terms: anamorelin, cachexia index, cancer anorexia, factor‐alpha, gastric cancer, interventions, models, and ono‐7643. This limited overlap confirms that these analyses measure very distinct phenomena.

**FIGURE 9:**
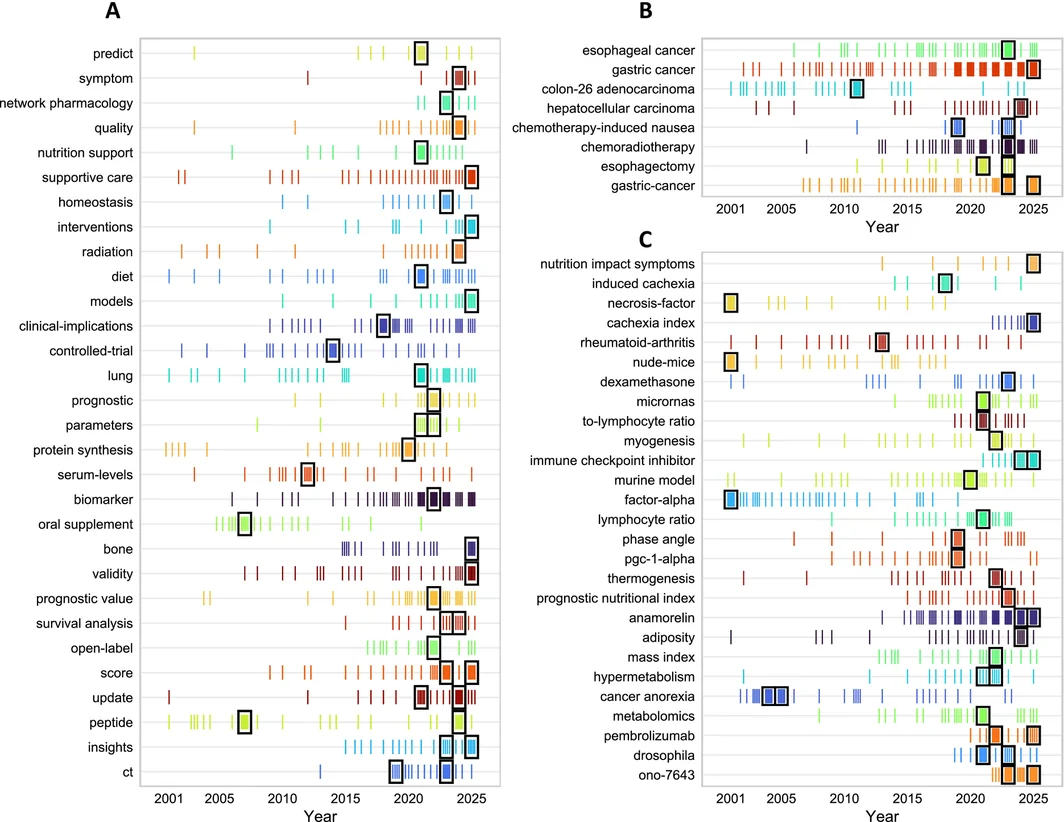
Burst analysis of keywords. The temporal event tick plot of keyword occurrences over time shows clusters of keyword usage that indicate a burst. Each tick mark indicates the use of a keyword and clusters that meet the threshold of a burst are indicated by boxes. For clarity, the ticks in a given year are slightly offset. Keywords were included if the frequency showed an increase of greater than five‐fold over an estimated baseline usage. Cluster analysis was used to identify three groups using AI‐embedding and LLM analysis provided the names and descriptions of each cluster shown in Table 3.

**TABLE 3:** Names and descriptions of the AI‐guided clusters for the burst analysis in Figure 9.

Cluster	Panel	Name	Description
1	A	Biomedical Prediction and Intervention	Related to predicting clinical outcomes, intervention strategies, and exploring various aspects of biomedical research and care
2	B	Gastrointestinal Cancer Treatments	Related to surgical and pharmacological interventions for various types of gastrointestinal cancers
3	C	Biomedical Metabolic Dysregulation	Related to metabolic dysregulation in various biomedical contexts such as cachexia, arthritis, immune checkpoint inhibitors, and nutrition impact symptoms

The Biomedical Prediction and Intervention cluster contains a number of very general terms. Bursts for nutritional support and diet both occur in 2021. A study with the keyword lung shows a burst in 2021 and a more recent burst in bone‐related research is seen in 2025. The term biomarker shows a wide range of usage, with a burst appearing in 2022.

The Gastrointestinal Cancer Treatments Cluster shows bursts in specific cancers starting in 2021. The keyword chemotherapy‐induced nausea experienced two bursts in 2019 and 2023. The earliest burst in this cluster is colon‐26 adenocarcinoma, which refers to a widely used murine model for cachexia [15].

The Biomedical Metabolic Dysregulation cluster reveals several bursts in keywords associated with inflammation. Lymphocyte‐ratio refers to a blood test for inflammation [16] peaks in 2021. The terms dexamethasone and Pembrolizumab are immunomodulatory therapies [17, 18]. Dexamethasone shows a burst in 2023 and Pembrolizumab shows bursts in 2022 and 2025. The more general term immune‐checkpoint inhibitor which describes the mechanism of action of Pembrolizumab shows bursts in 2024 and 2025. This suggests that cachexia research in immune therapy is experiencing a very recent burst in activity.

Activity in the area of metabolism is demonstrated by the bursts in several keywords. The master metabolic regulator PGC‐1α shows a burst in 2019. This protein is involved in mitochondrial biogenesis, energy metabolism, and thermogenesis [19]. The related terms thermogenesis show a burst in 2022 and hypermetabolism shows bursts in 2021 and 2022.

## 4 Discussion

For a long time, cachexia was considered merely a side effect of cancer with no clinical relevance; however, in recent decades, it has become clear that it is a fundamental driver of poor prognosis across a wide range of cancers [20]. Indeed, cancer cachexia directly contributes to reduced treatment tolerance, impaired functional status, diminished quality of life, and increased morbidity and mortality [21]. It is estimated that cancer cachexia is affecting 50%–80% of cancer patients [22, 23], with a more recent meta‐analysis suggesting a prevalence of approximately 33% [1]. Even at this lower prevalence, the projected 2 million new cancer cases expected in 2025 [24] would translate into a significant number of patients impacted by cachexia. In this analysis, we have shown that the field of cancer cachexia is rapidly growing with an annual growth rate during the period from 2001 to 2025 of 6.7% compared with the estimated overall growth rate of the scientific literature of 4.1% [12]. The formation of the Society for Sarcopenia, Cachexia and Wasting Disorders in 2008 and the formation of the Cancer Cachexia Society in 2017 certainly had an influence on the upward trajectory of research activity and publications.

Despite this promising growth, it must also be recognized that over this 25‐year period there have been less than 6000 papers on cancer cachexia (using the inclusion criteria of this study). In contrast, a search of WOSCC with the same search parameters found nearly 2.3 M papers on cancer and approximately 54 K papers on pancreatic cancer alone. Indeed, cancer cachexia continues to be one of the most underestimated and underrecognized medical consequences of malignant cancer [25]. This may be partly explained by the prioritization of cancer focus itself, the difficulty of diagnosing cachexia particularly in its early stages, and the challenge of setting a clear, standardized clinical definition.

It is evident that the most frequently chosen journal by the cancer cachexia research community is the *Journal of Cachexia, Sarcopenia and Muscle*, that published around 8% of all the cachexia related papers since the journal was established in 2010. Its popularity is likely influenced by its specific area of interest and the high impact factor, which contributes to its appeal, as reflected by its high H‐index and number of locally cited sources. In addition to journals focused on basic and preclinical science, several of the top journals have a strong clinical focus. Journals with an emphasis on the nutritional aspects of the syndrome are also prevalent. This emphasis could be expected as cancer cachexia is closely associated with malnutrition and undernutrition [26]. As defined in the international consensus paper by Fearon et al., the loss of skeletal muscle from cancer cachexia cannot be fully reversed by conventional nutritional support [2], but nutritional interventions are still included in clinical management. If mild nutritional disorders are not addressed early, they can progress into irreversible nutritional problems that complicate the clinical scenario [26, 27]. Especially in the early stages, maintaining an appropriate nutritional intervention is essential for possible recovery and improved quality of life [26, 28].

The network of collaboration plays an important role in the advancement of the field. The exchange of experiences, methodologies, and perspectives across disciplines is particularly critical in complex fields such as oncology. It has been shown that researchers tend to collaborate with peers in their proximity; however, research involving multiple institutions or international collaborations has been shown to have more success in generating high‐impact publications and receiving grant funding [29, 30].

A strong collaboration network contributes to the scientific community's collective capacity to solve complex problems and accelerate the translation of findings into clinical practice. In the context of the cancer cachexia research community, it is evident from Figure 4 that scientific collaboration tends to occur predominantly among geographically close researchers. This is shown by the presence of distinct communities in both the author and country networks. That being said, the network connections are indeed quite dense across the different communities, suggesting significant global collaborations. The cachexia community should continue to foster and prioritize these global collaborations to help accelerate new discoveries in clinical and basic research.

When considering the number of publications per country, the United States ranks first, followed by China and Japan. This distribution likely reflects both the size of these countries and the number of researchers engaged in cancer cachexia research. Notably, Italy and the UK, despite having smaller populations, demonstrate a proportionally high number of publications compared to the top three countries. Of particular interest, the United States and Italy show the highest number of multi‐country collaborations (MCPs), which may partly explain their elevated publication output in this field. In contrast, the United Kingdom ranks 5th in number of total publications but is 2nd in both total citations and citations per article. The work of the late UK author, Professor Kenneth Fearon (1945–2016), a globally recognized expert in cachexia, did not make the top 25 in terms of number of papers but was third in both local citations and H‐index. The collaboration network in Figure 5C clearly shows that the United States has the most branched and extensive collaborative network within the cachexia field. Notably, there is a close relationship and strong connection with several European countries such as Germany, the UK, and Italy. Interestingly, although well connected with other countries and regions, European countries form a close and cohesive network of collaborations. This may be due to simple geographical proximity, or perhaps the more interconnected relationships between universities and researchers. In this regard, the European Union sponsors several programs that support intra‐European research mobility, particularly for PhD students, postdocs, and researchers, which foster collaboration and development.

It is interesting to note that among the superset of most locally and globally cited papers shown in Figure 6, 10 of the 15 are related to the clinical definition, prediction, and management of cachexia. Several of these were published in the last 5 years, suggesting that the development of guidelines in these areas is still an active area of investigation.

In the top three papers is a recent study of the use of the humanized monoclonal antibody therapeutic, Ponsegromab, an inhibitor of the myokine GDF‐15 [31]. The last two papers in Figure 6 show relatively high global indices but are at or near zero in local citations. These papers report on the impact of extracellular vesicles in liver metabolic dysfunction [32] and the systemic impacts of COPD [33]. The high global impact and limited impact on the cachexia field suggest that knowledge from these studies may be interesting avenues for future cachexia research.

The co‐occurrence network revealed four communities of keywords which AI described as being related to (1) prognosis and body composition (2) cancer care and management (3) muscle metabolic regulation and (4) appetite regulation. These four descriptions aptly describe the overall landscape of cachexia research. In the muscle metabolic regulation community, two of the central keyword nodes are mice and cells. This could suggest that many of these metabolic and mechanistic studies are still in the preclinical stage. Translation of preclinical findings to clinical studies is indeed a very long process. The earliest papers on the impact of GDF‐15 in cancer cachexia came out in 2016 [34, 35] and the development of Ponsegromab was published 8 years later [31].

The trend and burst analyses shown in Figures 8 and 9 show distinct aspects of the progression of cachexia research focusing on longer term trends and more short‐term bursts in research respectively. The largest clusters in both analyses are associated with metabolism, therapeutic interventions, and clinical outcomes. It is interesting that the trend analysis revealed a distinct cluster associated with cytokines and inflammatory mediators. The burst analysis revealed a set of hotspots associated with immunometabolism, which are more recent than the median locations of the cytokine trends. It is reasonable to postulate that these earlier cytokine analyses set the foundation for the more recent bursts in immunotherapies.

The keywords anamorelin and Ono‐7643, the drug formulation of anamorelin, were among the few that were found in both the trend and burst analyses. Studies on these topics go back more than a decade. Clinical trials of Ono‐7643 (also called RC‐1291) go back to 2005 and some are still ongoing. The recent publication activities are likely associated with interest in the trial outcomes which found significant increases in body weight, lean body mass, and fat mass [36], but no significant changes in adverse event rates, overall survival, hand grip strength, and appetite [37].

There are some important limitations to bibliometric analyses that should be mentioned. The extraction of papers using the simple Boolean topics query “cachexia” and “cancer” may not find all of the relevant papers in this area, but the coverage should be sufficient to be highly representative of the field. The co‐occurrence, trend, and burst analyses are all based upon the superset of author reported keywords and the Keywords Plus generated by Web of Science. These terms provide a useful albeit incomplete representation of the contents of the paper. As shown in Table S1, the author provided keywords were missing in 21.1% of the papers and Keywords Plus were not computed for 3.6% of the papers, so the keyword data was not complete.

The use of AI in naming the co‐occurrence network communities and identifying and naming the clusters in the trend and burst analyses is a potential source of variability in the analyses. LLM‐generated results are nondeterministic and future evaluation of the same data may yield somewhat different names and descriptions. Therefore, the AI‐generated clusters and names should be considered interpretive summaries and not rigorously determined classifications. Importantly, the co‐occurrence network, trend and burst analyses were performed computationally and are independent of the subsequent AI‐based labeling.

## 5 Conclusions

This bibliometric analysis of the field of cancer cachexia over the last 25 years has shown that this is a rapidly growing and dynamic field of investigation. That said, it is still a relatively small field, and the research networks in terms of authors and institutions are still somewhat siloed based on geography.

The formation of the Society for Sarcopenia, Cachexia and Muscle Wasting in 2008 and the Cancer Cachexia Society in 2017 certainly had a positive impact on the field, as did the launch of the Journal of Cachexia, Sarcopenia and Muscle (JCSM) in 2010. The rapid growth of the number of papers and the high impact factor of JCSM, hovering around 10 for the last 5 years, has provided a focused forum for high caliber research in this area. Our analysis also revealed a trend toward an increased focus on the cytokines and inflammatory mechanisms, and burst analysis revealed recent hotspots in immunomodulatory therapeutic studies.

Bibliometric analyses provide a unique means to quantitatively analyze the progression of research in a particular area. The publication trends in terms of authors, journals, and countries provide a high‐level view of the activities in the field. The scientific details of the research are currently based only on keywords or the Keywords Plus. Although useful, the reliance on this limited set of information limits the detail that can be derived from these studies. The ongoing development of bibliometric methods that utilize natural language processing and AI methods to extract more detailed information will be a significant step forward in adding more detail to bibliometric studies [38, 39]. Continued advances in the application of AI in bibliometrics are also accelerating, which should further enrich the details available from bibliometric studies. We hope that the quantitative analysis of the field of cachexia presented here provides an informative and actionable guide to the past, present, and potential future directions of this critically important field.

## Author Contributions

**Thomas M. O'Connell:** conceptualization, investigation, writing – original draft, writing – review and editing, visualization, project administration, software, formal analysis, supervision, data curation. **Radhika Gedela:** software, formal analysis, visualization, writing – review and editing, data curation, methodology, investigation, conceptualization. **Fabrizio Pin:** writing – original draft, writing – review and editing, investigation.

## Funding

The authors have nothing to report.

## Conflicts of Interest

The authors declare no conflicts of interest.

## Supporting information


**Data S1:** AI prompt code for community and cluster naming.docx.

**Data S2:** Full Bibliometrix Dataset.csv. Full set of information on all 5775 references extracted from Bibliometrix.

**Data S3:** WOSCC Cancer Cachexia Results 092425.txt. This is the raw data on all 5775 references collected from the WOSCC search that is used in all subsequent analyses.

**Figure S1:** Annual scientific production between 2001 and 2025. The data has been log transformed and the red line shows the fit to linear function with *R* ^2^ value = 0.95.

**Table S1:** Completeness of metadata from full WOSCC data extraction.

**Table S2:** Citation metrics for all 5775 articles used in this analysis.

## Data Availability

The raw WOSCC Data S3 and the Bibliometrix processed Data S2 are available as supporting information. The individual datasets extracted from the publication dataset used to generate the figures have been saved and are available upon request.
