# Supplementary material for: The Growth and Trends in Cancer‐Induced Cachexia Research: A Bibliometric and AI‐Guided Visualization Analysis
Source: Cancer Rep (Hoboken). 2026 Oct 1;9(10):e70690. doi: 10.1002/cnr2.70690 (PMC13628286; doi:10.1002/cnr2.70690)
Supplement: Supplementary file 1 [file CNR2-9-e70690-s003.docx]

\# ****************** Function to ask AI for a community name

name_community <- function(terms) {

prompt <- paste(

"I have a community of biomedical keywords produced by semantic clustering.",

"Please do two things:",

"1. Propose a short cluster name of 2 to 5 words.",

"2. Provide a one-sentence explanation.",

"",

"Keywords:",

paste(terms, collapse = ", "),

"",

"Return your answer in exactly this format:",

"Cluster name: <name>",

"Explanation: <one sentence>"

)

response <- openai::create_chat_completion(

model = "gpt-3.5-turbo",

messages = list(

list(

role = "system",

content = "You are an expert in biomedical bibliometrics and scientific topic classification."

),

list(

role = "user",

content = prompt

)

)

)

\# For openai 0.4.1, choices is typically flattened

if (is.data.frame(response$choices)) {

return(response$choices$message.content[1])

}

\# Fallback for other possible structures

if (is.list(response$choices) &&

length(response$choices) >= 1 &&

is.list(response$choices[[1]]) &&

!is.null(response$choices[[1]]$message$content)) {

return(response$choices[[1]]$message$content)

}

stop("Unrecognized response structure. Run str(response) to inspect it.")

}

\# Get terms for each cluster

community_terms <- keywords %>%

group_by(community) %>%

summarise(terms = list(Term), .groups = "drop")

\# Ask AI to name each cluster

community_terms <- community_terms %>%

rowwise() %>%

mutate(ai_label = name_community(terms)) %>%

ungroup()

print(community_terms$ai_label)
